# A Case of Diabetes Mellitus Treated with Chloral Hydrate—Relieved

**Published:** 1877-03-20

**Authors:** E. Cross


### A CASE OF DIABETES MELLITUS TREATED WITH CHLORAL HYDRATE; RELIEVED.

BY E. CROSS, M. D.

Last fall, Mr. N., a Hebrew merchant of this place, aged thirty-two, called on me stating that for the past twelve months he had been suffering from what his physician called diabetes, and that it had been pronounced incurable. The patient stated that he was drinking from six to ten pints of water every day and perhaps twice as much at night, and that he was forced to arise so often at night to void his urine and procure drink as to deprive himself and room-mate of necessary sleep.

The patient was of constipated habit, with a worn and anxious expression, mucous membrane of mouth dry, tongue red. A specimen of the urine was obtained and submitted to my partner, Dr. E. T. Easley, for analysis, who found its specific gravity to be as high as 1050, and yielding ten per cent. sugar. Upon enquiry I ascertained that his treatment had been such as is usually prescribed, diet, flannel, phosphoric acid, opium, etc., all of which had failed to give relief. At this time his emaciation was so rapid, and his thirst and nervousness from want of sleep so great that he was almost forced to give up business.

Proposing to begin treatment by obtaining a good night's rest for my patient, I directed twenty grains chloral every half hour until sleep could be induced. The first night he took four doses, and reported rest and more sleep than he had had for six months, adding that he had only passed water every hour instead of every half hour as he had previously done. I ordered the treatment continued through the day, the same dose every three hours, and at night as before. In a few days marked improvement was evident. The chloral was continued for four weeks, with the addition of 25 drops muriated tincture of iron three times daily, when he reported himself as cured ; said he slept well, that he did not get up oftener than twice at night, no thirst, urine nearly normal and he was gaining in flesh. About this time he called at my office to inqure about taking a trip across the plains, and was advised to go. He returned after four months a well man, and to-day, twelve months from the time treatment began, is in as perfect health as ever.

I wish to call the attention of the profession to this case and ask them, at least, to give the remedy a trial. It can do no harm, and may do good, at any rate, I should like others to make further experiments with it. If the trouble be dependent upon nervous disturbance, without organic lesion, why may not this agent be useful? I would be pleased if the profession should give chloral a trial, for unquestionably its action was highly satisfactory in the only case in which I have used it.—*Clin. Record.*

